# Dendritic cells combined with tumor cells and α-galactosylceramide induce a potent, therapeutic and NK-cell dependent antitumor immunity in B cell lymphoma

**DOI:** 10.1186/s12967-017-1219-3

**Published:** 2017-05-26

**Authors:** Laura Escribà-Garcia, Carmen Alvarez-Fernández, Marta Tellez-Gabriel, Jorge Sierra, Javier Briones

**Affiliations:** 1 Hematology Service, Hospital de la Santa Creu i Sant Pau, Mas Casanovas 90, 08041 Barcelona, Spain; 2 Laboratory of Experimental Hematology-IIB, Institut Recerca Hospital de la Santa Creu i Sant Pau, Barcelona, Spain; 3 Autonomous University, Barcelona, Spain

**Keywords:** Immunotherapy, Dendritic cells, iNKT cells

## Abstract

**Background:**

Invariant natural killer T (iNKT) cells are a small population of lymphocytes with unique specificity for glycolipid antigens presented by non-polymorphic CD1d receptor on dendritic cells (DCs). iNKT cells play a central role in tumor immunology since they are implicated in the coordination of innate and adaptive immune responses. These cells can be activated with the prototypic lipid α-galactosylceramide (α-GalCer), stimulating interferon gamma (IFN-γ) production and cytokine secretion, which contribute to the enhancement of T cell activation.

**Methods:**

We evaluated the antitumor effect of a combination of dendritic cells (DCs) and tumor cells with the iNKT cell agonist α-GalCer in a therapeutic model of B cell lymphoma. iNKT, NK and T cell phenotype was determined by flow cytometry. Serum cytokines were analyzed by Luminex technology. Significant differences between survival curves were assessed by the log-rank test. For all other data, Mann–Whitney test was used to analyze the differences between groups.

**Results:**

This vaccine induced a potent (100% survival), long-lasting and tumor-specific antitumor immune response, that was associated with an increase of both Th1 cytokines and IFN-γ secreting iNKT cells (4.59 ± 0.41% vs. 0.92 ± 0.12% in control group; p = 0.01) and T cells (CD4 IFN-γ^+^: 3.75 ± 0.59% vs. 0.66 ± 0.18% p = 0.02; CD8 IFN-γ^+^: 10.61 ± 0.84% vs. 0.47 ± 0.03% p = 0.002). Importantly, natural killer (NK) cells played a critical role in the antitumor effect observed after vaccination.

**Conclusions:**

This study provides clinically relevant data for the development of iNKT-cell based immunotherapy treatments for patients with B cell malignancies.

## Background

Invariant natural killer T (iNKT) cells are a small population of lymphocytes characterized by the expression of an invariant T cell receptor (TCR) encoded by Vα14Jα18 and Vβ8 segments in mice, and Vα24Jα18 and Vβ11 segments in humans [1–3]. These cells have a unique specificity for numerous endogenous and exogenous glycolipid antigens presented by the non-polymorphic CD1d receptor on antigen presenting cells (APCs) [1, 2, 4].

iNKT cells play a central role in tumor immunology since they coordinate innate and adaptive immune responses and can be activated using the synthetic glycolipid α-galactosylceramide (α-GalCer) [1, 2, 5, 6]. The interaction between CD1d-glycolipid complex and the invariant TCR of iNKT cells stimulates interferon gamma (IFN-γ) production and the secretion of a large number of other cytokines (e.g. IL-12, IL-4, IL-17) that promote tumor eradication [7, 8]. In addition, iNKT cell activation contributes to the enhancement of dendritic cell (DC) function and the activation and expansion of NK cells [2, 9] and antigen-specific B and T cells [6].

The capacity of iNKT cells to induce potent innate and antigen-specific immune responses [1, 2, 5, 10] provides the basis for designing an effective immunotherapy to enhance immune responses against tumors. Different iNKT cell-directed therapies has been studied so far, including administration of iNKT cell-activating ligands such as α-GalCer, and the administration of DCs or tumor cells loaded with this glycolipid [7, 11–14]. Activation of iNKT cells by giving soluble free α-GalCer in vivo has been shown to induce potent antitumor responses in some murine tumor models [11], although it induces a long-term iNKT cell anergy causing unresponsiveness to sequential stimulation with that glycolipid [15, 16]. When iNKT cells are activated with α-GalCer, the interaction of iNKT cells with APCs seems to be a key factor for the development of antitumor activity. Previous studies in murine models suggested that injection of DCs loaded with α-GalCer induces prolonged cytokine responses with an enhancement of antitumor effect compared with injection of free α-GalCer [7, 12]. Additional studies showed that tumor B cells loaded with α-GalCer induced a potent antitumor immunity as a prophylactic treatment [13, 14].

Although these different strategies resulted in promising data in pre-clinical studies their translation to the clinical setting proved to be less effective. α-GalCer was tested in a clinical trial with solid cancer patients and only transient iNKT cell activation was detected in a minority of patients [17, 18]. Other clinical trials in different solid cancer and myeloma patients were carried out using α-GalCer-loaded DCs and, while most of the patients showed an increase of IFN-γ and IL-12 serum levels, no antitumor responses were noted [10, 19–22].

The lack of clinically relevant antitumor efficacy of α-GalCer or DCs loaded with α-GalCer strategies prompted to search for different approaches. We reasoned that the activation of iNKT cells in the presence of DCs, α-GalCer and tumor cells, as an antigen source, would translate into a highly effective immunotherapy treatment. Hence, we evaluated the antitumor effect of a vaccine that combines DCs and irradiated tumor cells with the iNKT cell agonist α-GalCer in a B cell lymphoma mouse model. We show that this approach induces a strong cytokine production and activation of NK, B and T cells and, more importantly, a potent antitumor efficacy in a therapeutic setting. Our results further support the use of a combination of DCs and α-GalCer mixed with tumor cells as a therapeutic treatment against B cell lymphoma.

## Methods

### Mice

All experiments and care of animals were conducted according to European Animal Care guidelines and approved by the Ethical Committee of Animal Experimentation at Hospital de la Santa Creu i Sant Pau. Female Balb/c mice in age from 6 to 7 weeks were obtained from Charles River (France). They were acclimatized for 1 week and then were housed under specific pathogen-free conditions at the Laboratory Animal Facility in accordance with the animal welfare guidelines set by Ethical Committee of Animal Care of Hospital de la Santa Creu i Sant Pau (Barcelona). Mice were housed under the following conditions: temperature of 21.5 °C, humidity of 60%, and 12 h light/dark rhythm. All mice were daily observed by the animal investigators for survival, with more intensive revision on days 1, 2, 3 and 5 and after tumor challenge (two times a day). Humane endpoints of mice included weight loss of 20–25%, weakness preventing them from obtaining food or water, loss of appetite (anorexia for 24 h), moribund state, or unable to participate in normal activities due to the tumor growth. All mice were euthanized by decapitation under anesthesia when two or more of these humane endpoints were observed.

### Cell lines

4TOO is a Balb/c plasmacytoma cell line expressing major histocompatibility complex (MHC) class I H-2^d^ molecules gently provided by Dr. M. Khuel (NCI, Bethesda, MD). A20, a syngeneic Balb/c B cell lymphoma expressing MHC class I and II H-2^d^ molecules, was obtained from the American Type Culture Collection (ATCC, USA). Tumor cells were maintained in complete medium consisting of RPMI 1640 supplemented with 10% heat-inactivated fetal calf serum (FCS), 100 U/ml penicillin, 100 μg/ml streptomycin and 50 μM β-2-mercaptoethanol (Life Technologies, USA). Cells were grown in suspension culture at 37 °C in 5% CO_2_.

### B cell lymphoma tumor models

4TOO and A20 tumor cells were thawed from a common frozen stock and grown in vitro in complete medium for 3 days before use. On the day of tumor injection, cells were washed with complete medium and diluted to the appropriate concentration in phosphate-buffered saline (PBS) solution. Mice (8–10/group) were inoculated intravenously (iv) with 4 × 10^5^ 4TOO tumor cells or subcutaneously (sc) with 1 × 10^6^ A20 tumor cells in a volume of 0.1 ml.

### Generation of DCs

DCs were generated from murine bone marrow as previously described [23]. Briefly, bone marrow from Balb/c mice was flushed from the long bones (femur and tibia) and depleted of red blood cells by 3 min incubation with erythrocyte lysis solution (BD Pharm Lyse Buffer, BD Bioscience, USA). Cells were washed and cultured in complete medium plus 20 ng/ml of recombinant murine granulocyte–macrophage colony-stimulating factor (rm-GM-CSF; Peprotech, USA). On day 5 of culture, DCs were collected from the loosely adherent population and replated with fresh complete medium plus rmGM-CSF. After 48 h, cells were used for vaccine generation.

### In vivo vaccination experiments

The therapeutic vaccine is composed by DCs and 4TOO tumor cells (total of 10^6^ cells, ratio 1:1), which were irradiated 30 Gy immediately before injection, combined with 2 μg/mouse of α-GalCer (KRN7000; Enzo Life Sciences, USA) added to the cell suspension prior to mice treatment (referred to as Mix + GalCer vaccine). The therapeutic treatment consisted in a single iv injection of the vaccine 2 days after tumor challenge (8–10 mice/group). Animals were daily followed for survival.

In some experiments, surviving mice from the first tumor challenge (i.e., that were tumor-free after 100 days) received either a second identical lethal tumor inoculation or were injected sc with the syngeneic A20 B-cell lymphoma.

### In vivo cell depletion experiments

Mice (5/group) were treated with the vaccine 2 days after 4TOO tumor challenge. Specific groups of mice were depleted for CD4^+^, CD8^+^ or NK cells by intraperitoneal (ip) injection of anti-mouse CD4 monoclonal antibody (mAb) (150 μg, GK1.5 clone; BioXCell, USA), anti-mouse CD8 mAb (500 μg, 53-6.72 clone; BioXCell, USA) and anti-asialo GM1 (10 μl, Wako Chemicals, Germany) antibodies. These antibodies were injected on days −2, −1 and 1, relative to tumor inoculation, and then weekly for 2 months. A group of tumor-bearing mice and Mix + GalCer treated mice received the rat IgG2 antibody (2A3 clone; BioXCell, USA) as a control. The efficacy of these depleting antibodies were validated by flow cytometry analysis of splenocytes using anti-mouse mAbs against CD4 (RM4-4) and CD8β (H35-17.2) (both from eBiosciences, USA) for T cells, and NKp46 (29A1.4.9) and CD3 (145-2C11) (both from Miltenyi Biotec, Germany) for NK cells. All antibodies depleted more than 95% of the specific population while the other subsets, including iNKT cells, remained within similar levels to controls.

### Flow cytometry studies

Anti-mouse mAbs against CD4 (GK1.5) and CD8α (53-6.72) (both from Miltenyi Biotec, Germany) were used to identify T cells and anti-mouse CD3 (145-2C11) and NKp46 (29A1.4.9) antibodies (both from Miltenyi Biotec, Germany) were used for NK cell analysis. Unloaded and α-GalCer analogue (PBS-57)-loaded CD1d tetramers were kindly provided by the NIH Tetramer Core Facility (Atlanta, GA) and were used for iNKT cell detection (1.2 μg for 10^6^ cells in 100 μl), together with an anti-mouse TCRβ antibody (REA318; Miltenyi Biotec, Germany). Cells were stained for 30 min at 4 °C in PBS containing 1% BSA and 0.01% NaN_3_ (staining buffer). Data was acquired on a MACSQuant Analyzer 10 (Miltenyi Biotec, Germany) and analyzed using FlowJo Version 10 software (TreeStar, USA).

### Intracellular IFN-γ detection

For intracellular staining of IFN-γ in NK and iNKT cells, splenocytes were fixed with 1.5% formaldehyde solution during 10 min and permeabilized with 0.5% Tween-20 solution during 15 min, both incubations at 4 °C. Cells were stained using the anti-mouse IFN-γ mAb (AN.18.17.24; Miltenyi Biotec, Germany), for 30 min at 4 °C. For T cell intracellular staining of IFN-γ, splenocytes were cocultured with irradiated (30 Gy) 4TOO tumor cells in a 2:1 effector:target (E/T) ratio for 24 h. Brefeldin A was added to the cells 4 h before IFN-γ detection to blocked cytokine secretion. Cells were fixed, permeabilized and stained for IFN-γ detection as described above. All data was acquired on a MACSQuant Analyzer 10 (Miltenyi Biotec, Germany) and analyzed using FlowJo Version 10 software (TreeStar, USA).

### Detection of serum cytokines

Levels of IFN-γ, IL-12p70, TNF-α, IL-4 and IL-17 in mice sera were detected using Luminex bead array (ProcartaPlex, eBioscience, USA) according to manufacturer’s instructions. Acquisition was performed on a LUMINEX^®^ 100/200™ analyzer (Luminex). Data analysis was performed using ProcartaPlex Analyst 1.0 (eBioscience, USA).

### Indirect immunofluorescence assay for detection of serum antibodies

Three microliters of serum samples from naïve, untreated and Mix + GalCer treated mice were incubated with 4TOO tumor cells (3 × 10^5^ cells) in 50 μl of staining buffer during 30 min at 4 °C. Cells were washed and incubated with a PE-conjugated anti-mouse IgG polyclonal antibody (eBioscience, USA) for 30 min at 4 °C. Controls included 4TOO tumor cells with no serum, A20 tumor cells incubated with the different sera and both cell lines with naïve serum incubation. Relative IgG levels were obtained by dividing the mean fluorescence intensity (MFI) of treated serum samples by the MFI of naïve serum control.

### Statistical analysis

Results are expressed as the mean ± SEM. Kaplan–Meier plots were used to analyze mice survival and the significant differences between survival curves were assessed by the log-rank test. For all other data, Mann–Whitney test was performed to analyze the differences between groups. All statistical analysis and graphics were performed using GraphPad Prism 6 (GraphPad Software, USA). p values <0.05 were considered significant.

## Results

### Therapeutic treatment with Mix + GalCer vaccine exhibits a potent in vivo antitumor effect

**Fig. 1:**
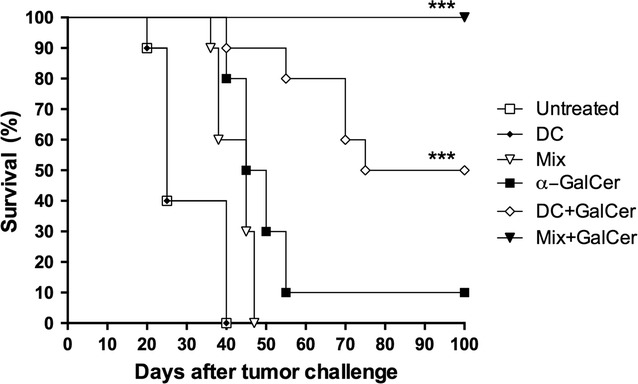
Mix + GalCer vaccine induces a potent in vivo therapeutic antitumor immunity against B cell lymphoma. Balb/c mice (n = 10) were injected with 4 × 10^5^ 4TOO tumor cells (iv) on day 0 and were treated 2 days later with a single dose of Mix + GalCer vaccine (iv) or control treatments, including α-GalCer alone, DCs alone, DCs with α-GalCer and Mix. Mice were monitored daily for survival. Data represents overall survival of one of three independent experiments. ***p < 0.0005

First, we studied the capacity of a single dose of Mix + GalCer vaccine to stimulate antitumor immunity against B cell lymphoma in a therapeutic setting. For this purpose, mice (n = 10) were treated with the vaccine (iv) 2 days after 4TOO tumor challenge. In addition, other vaccine combinations were tested as controls including DCs alone, α-GalCer alone, DCs with α-GalCer, and DCs with tumor cells (Mix). No significant antitumor immunity was observed in mice treated with DCs alone, Mix or free α-GalCer (0, 0 and 10% survival, respectively) (Fig. 1). The most effective combination was the Mix + GalCer vaccine, resulting in long-term survival of 100% of mice (p = 0.001), while the combination of DCs with α-GalCer exhibited a lower antitumor effect (50% survival; p = 0.001).

### Mix + GalCer vaccine confers long-lasting and tumor-specific immunity

**Fig. 2:**
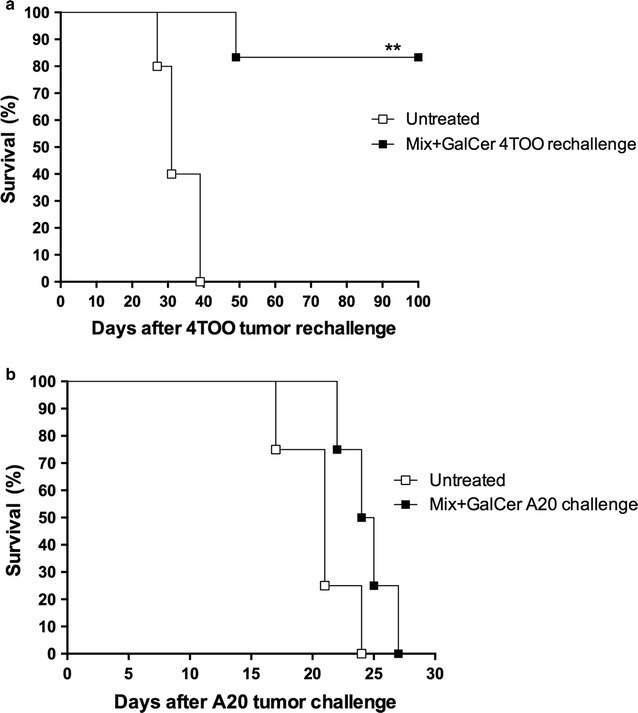
Mix + GalCer vaccine confers long-lasting and tumor-specific immunity. **a** Mix + GalCer treated mice that survived from the first 4TOO tumor challenge (n = 6) were reinjected with 4TOO tumor cells again (4 × 10^5^ cells/mice, iv). Untreated age-matched mice were injected with 4 × 10^5^ 4TOO tumor cells (iv). **b** Mice treated with Mix + GalCer vaccine that survived after the first 4TOO tumor challenge (n = 5) were injected with the syngeneic A20 B-cell lymphoma (1 × 10^6^ cells, sc). Untreated age-matched mice were injected with 1 × 10^6^ cells A20 tumor cells (sc). **p = 0.005

To test whether treatment with Mix + GalCer vaccine could induce long-term immunity against the tumor, a group of animals (n = 6) that survived after the first tumor injection were challenged again with 4TOO tumor cells. Five of six immunized mice remained tumor-free 100 days after rechallenge (p = 0.005), whereas all control animals (n = 10) were not able to reject the tumor (Fig. 2a).

In addition, immunized mice (n = 5) received a syngeneic A20 B-cell lymphoma challenge 100 days after the 4TOO tumor injection. In this case, all mice died due to lymphoma, suggesting that the immune response induced by the Mix + GalCer treatment was tumor-specific (Fig. 2b).

### Therapeutic vaccination increases the iNKT and NK cell populations in vivo

**Fig. 3:**
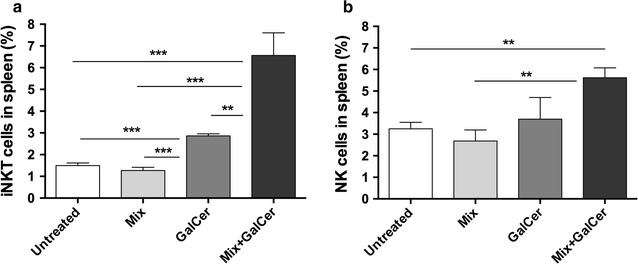
Mix + GalCer treatment induces an increment of iNKT and NK cells. **a** Percentage of iNKT cells in spleen 3 days after injection of Mix, α-GalCer or Mix + GalCer treatment (n = 4 per group). iNKT cells were analyzed by flow cytometry and gated as TCRβ^+^ and Tet-PBS57^+^ cells. **b** Percentage of NK cells in spleen 3 days after Mix, α-GalCer or Mix + GalCer treatment (n = 2 per group), analyzed by flow cytometry as CD3^−^ NKp46^+^ cells. **p < 0.005; ***p < 0.0005

Both iNKT and NK cells were enriched in the spleen of Mix + GalCer treated mice after vaccination. Regarding iNKT cells, there was a moderate increment in mice treated with free α-GalCer (2.86 ± 0.06% vs. 1.5 ± 0.05% in control group; p = 0.0002), but they were further increased after Mix + GalCer treatment in comparison with α-GalCer vaccination (6.56 ± 0.46% vs. 2.86 ± 0.06% respectively; p = 0.005) and Mix treated mice (6.56 ± 0.46% vs. 1.27 ± 0.08%; p = 0.0001) (Fig. 3a). NK cells also exhibited a significant increment after Mix + GalCer treatment compared to Mix treated group (5.21 ± 0.28% vs. 2.68%; p = 0.003) and also showed a trend to an increment compared to α-GalCer treated mice (5.21 ± 0.28% vs. 3.69 ± 0.71%; p = 0.06) (Fig. 3b).

### Mix + GalCer treatment induces a combination of Th1, Th2 and Th17 cytokine profile

**Fig. 4:**
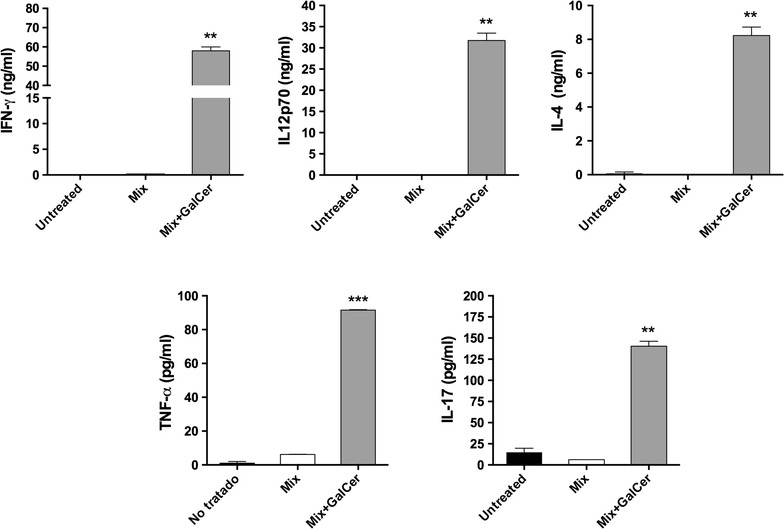
Mix + GalCer therapeutic vaccination induces an increment of serum cytokine levels. Serum samples of control group, Mix treated mice and Mix + GalCer treated mice (n = 2 per group) were analyzed for cytokine levels of IFN-γ, IL12p70, TNF-α, IL-4 and IL-17. Samples were obtained 12 h after treatment injection. **p < 0.005; ***p < 0.0005

We assessed the capacity of Mix + GalCer vaccine to induce IFN-γ, IL-12, tumor necrosis factor alpha (TNF-α), IL-4 and IL-17 cytokine secretion (Fig. 4). Mix + GalCer treated mice had a dramatic increase of IFN-γ and IL-12 serum levels, compared to mice treated with Mix (IFN-γ: 58.0 ± 2.0 ng/ml vs. 0.03 ± 0.002 ng/ml, p = 0.001; IL-12: 31.5 ± 1.75 ng/ml vs. 0.001 ± 0.0004, p = 0.003). In addition, there was a significant increase of TNF-α secretion after Mix + GalCer treatment in comparison with Mix treated mice (91.52 ± 0.3 pg/ml vs. 6.23 ± 0.03 pg/ml; p = 0.0001). We also observed higher amounts of IL-4 after Mix + GalCer vaccination (8.2 ± 0.5 ng/ml vs. 0.001 ± 0.0004 ng/ml in Mix treated mice; p = 0.003) and, interestingly, these mice also exhibited increased IL-17 serum levels in comparison with Mix treated group (140.4 ± 5.7 pg/ml vs. 6.2 ± 0.02 pg/ml; p = 0.001).

### iNKT, NK and T cells contribute to IFN-γ production after vaccination

**Fig. 5:**
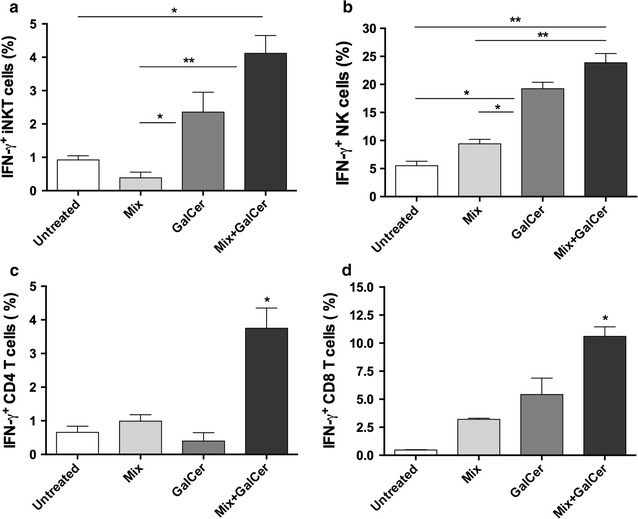
IFN-γ producing iNKT, NK and T cells are increased after Mix + GalCer vaccination. Untreated, Mix, α-GalCer and Mix + GalCer treated mice (n = 2 per group) were sacrificed 3 days after treatment. Splenocytes were assessed for IFN-γ secreting **a** iNKT cells or **b** NK cells by flow cytometry. In addition, 2 × 10^6^ splenocytes of each group were cocultured with irradiated 4TOO tumor cells (2:1 ratio) for 24 h. After this time, CD4^+^ (**c**) and CD8^+^ (**d**) T cells were assessed for IFN-γ secretion by flow cytometry. *p < 0.05; **p < 0.005

Animals treated with Mix + GalCer vaccine presented a high level of IFN-γ in serum suggesting that it might be a relevant cytokine in the antitumor immune response. Next, we studied which cells were involved in the systemic production of IFN-γ after treatment. Mix + GalCer treated mice had significantly higher amounts of IFN-γ producing iNKT cells in spleen, compared to untreated mice (4.59 ± 0.41% vs. 0.92 ± 0.12%; p = 0.01) (Fig. 5a). Moreover, the therapeutic treatment induced a trend to an increase of these cells compared to α-GalCer vaccination (4.59 ± 0.41% vs. 2.35 ± 0.59%; p = 0.09). Concerning IFN-γ secreting NK cells, α-GalCer treated mice exhibited an important increase of these cells compared to untreated animals (19.23 ± 1.17% vs. 5.50 ± 0.80%; p = 0.01). However, Mix + GalCer vaccine was the combination that induced the highest increment of IFN-γ secreting NK cells (23.87 ± 1.64% vs. 5.50 ± 0.80% in untreated mice; p = 0.003), with no statistical differences with α-GalCer treatment (23.87 ± 1.64% vs. 19.23 ± 1.17%; p = 0.1) (Fig. 5b).

Additionally, we also analyzed the presence of specific IFN-γ secreting CD4^+^ and CD8^+^ T cells in animals treated with Mix + GalCer vaccine. In this study, splenocytes were cocultured together with irradiated 4TOO tumor cells and we observed that the percentage of specific IFN-γ producing CD4^+^ and CD8^+^ T cells was significantly higher after Mix + GalCer vaccination, compared to untreated mice (CD4 IFN-γ^+^: 3.75 ± 0.59% vs. 0.66 ± 0.18% p = 0.02; CD8 IFN-γ^+^: 10.61 ± 0.84% vs. 0.47 ± 0.03% p = 0.002) and α-GalCer treated animals (CD4 IFN-γ^+^: 3.75 ± 0.59% vs. 0.4 ± 0.25% p = 0.02; CD8 IFN-γ^+^: 10.61 ± 0.84% vs. 5.41 ± 1.46% p = 0.04) (Fig. 5c).

### NK cells are critical for the therapeutic effect of the Mix + GalCer vaccine

**Fig. 6:**
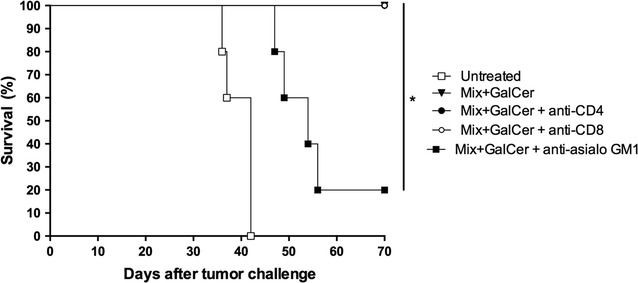
Effect of in vivo depletion of NK, CD4^+^ or CD8^+^ T cells after Mix + GalCer treatment. Mice (n = 5) were injected with 4TOO tumor cells (4 × 10^5^ cells, iv) and were treated 2 days after tumor injection with a single dose of Mix + GalCer vaccine (iv). Other groups of mice (n = 5 per group) received the same regimen of tumor and Mix + GalCer vaccine injection but they were depleted for CD4 (anti-CD4, GK1.5), CD8 (anti-CD8, 53-6.72) and NK (anti-asialo GM1) cells. *p = 0.01

We next determined which effector cells were involved in the in vivo antitumor effect of Mix + GalCer treatment. For this purpose, NK, CD4^+^ and CD8^+^ T cells were depleted using specific monoclonal antibodies. The presence of NK cells was found to be critical for the antitumor efficacy induced by Mix + GalCer vaccine since 80% of NK-cell depleted mice were unable to reject the tumor (p = 0.01 compared to treated mice with no depletion), whereas neither CD4^+^ nor CD8^+^ T cell depletion had a deleterious effect in the antitumor efficacy of the vaccine (100% survival) (Fig. 6).

### Therapeutic administration of Mix + GalCer vaccine induces a specific humoral immune response

**Fig. 7:**
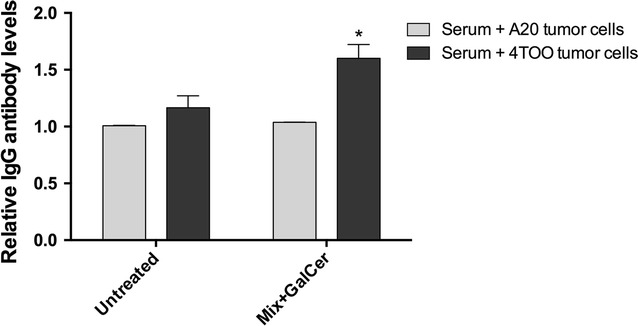
Mix + GalCer vaccine induces specific IgG antibodies that recognize 4TOO tumor cells. 4TOO and A20 tumor cells were incubated with serum samples of naive, untreated and Mix + GalCer treated groups and the relative specific IgG antibody levels were analyzed by flow cytometry (n = 3 per group) and normalized using the value of naive serum samples. *p < 0.05

To look for the generation of a humoral immune response after Mix + GalCer vaccination, serum samples from treated mice were incubated with 4TOO tumor cells and the presence of specific immunoglobulin G (IgG) anti-tumor antibodies were detected by indirect immunofluorescence. Specific anti-tumor antibodies were detected in the serum 14 days after Mix + GalCer injection, whereas untreated mice did not have IgG antibodies reacting against 4TOO tumor cells (1.60 ± 0.12 vs. 1.05 ± 0.06 relative IgG antibody levels; p = 0.04). Importantly, those IgG antibodies from treated mice were not able to recognize a syngeneic B cell lymphoma (i.e., A20), strongly suggesting that the humoral response generated was tumor specific (Fig. 7).

## Discussion

iNKT cell activation represents an attractive approach for cancer immunotherapy since iNKT cell stimulation induces a potent innate and adaptive immune system activation that can generates antitumor immunity [1, 2, 5, 10]. Here, we show that a single dose of a vaccine that combined DCs, tumor cells and α-GalCer (Mix + GalCer vaccine) is very effective in eradicating B cell lymphoma in vivo as a therapeutic treatment. Although other studies show that the combination of these three elements, together with the inhibition of regulatory T cells (Tregs) [24] are a good strategy to induce antitumor immune responses, the data presented here demonstrated that just the combination of DCs, tumor cells and α-GalCer prior to injection into tumor-bearing mice can induce the strongest antitumor effect. Our therapy generates a specific, memory and global immune system activation that is able to eradicate B cell lymphoma in vivo. The importance of combined DCs and α-GalCer with stressed tumor cells to improve the specificity and the potency of the antitumor immune response is clearly reflected in the highly percent of tumor-free mice after Mix + GalCer treatment, in comparison with those receiving only DCs and α-GalCer. The addition of tumor cells into the vaccine can induce T and iNKT cell responses toward a high range of MHC class I and II, and CD1d-related tumor antigens, including those antigens which are immunogenic but less representative. Of interest, the administration of α-GalCer alone in our model did not have any antitumor efficacy, in contrast to other studies which showed a considerable antitumor effect [7, 11]. The reduced antitumor efficacy that we observed in our B cell lymphoma model using α-GalCer alone mostly resembles the clinical scenario, where patients treated with this glycolipid did not experiment any antitumor effect although they had a moderate iNKT cell expansion [10, 17, 19, 20].

Moreover, in line with other studies, we observed that a single-dose of Mix + GalCer vaccine induced a significant increase of iNKT cells in the spleen that was greater than the one induced by α-GalCer alone [13]. Interestingly, NK cells had a similar kinetic behavior as iNKT cells, showing a greater increase in the spleen of mice treated with Mix + GalCer vaccine compared to α-GalCer alone. It is possible that the significant expansion of NK cells induced by Mix + GalCer vaccine may be related to iNKT cell activation, in line with data from previous studies [9].

Mix + GalCer vaccine was also able to protect mice from a second tumor challenge, indicating the generation of established adaptive memory immunity against tumor cells, and that it was tumor-specific. Previous studies using DCs or tumor cells loaded with α-GalCer exhibited similar long-lasting immunity in mice models of myeloma and B-cell lymphoma [13, 25], as well as in other non-hematological tumor models [14, 26]. In fact, iNKT cells are able to improve the generation and proliferation of CD4^+^ and CD8^+^ memory T cells [27], as well as to enhance B cell function and memory in mice [28].

Once analyzed the in vivo antitumor effect of Mix + GalCer treatment, we observed that the cytokine profile induced by the therapeutic vaccine was a combination of Th1, Th2 and Th17-type responses, which is consistent with a potent iNKT cell activation [1, 5]. The cytokine that showed the highest level in Mix + GalCer treated mice was IFN-γ, suggesting a critical involvement in the antitumor effect. In line with previous studies, high IFN-γ production is the main common characteristic in α-GalCer based treatments [14, 29]. In our study, we detected a significant increase of IFN-γ secreting iNKT, NK after Mix + GalCer injection, suggesting that all of these cells are involved in the IFN-γ production observed after treatment. Interestingly, in contrast with α-GalCer treatment, we observed that Mix + GalCer vaccine induced the generation of tumor-specific IFN-γ CD4 and CD8 T cells, which can be important to increase the levels of IFN-γ and to improve the antitumor effect and the generation of the memory anti-tumor immune response. Nevertheless, a definitive role of IFN-γ in the antitumor efficacy observed in our tumor model could only be demonstrated using IFN-γ knock-out mice.

In addition, there was a significant increase of IL-12 after Mix + GalCer therapy which may reflects the involvement of DCs in the generation of the antitumor immune response [14, 29]. Very interestingly, Mix + GalCer treated mice showed very high levels of IL-17 in their sera. It is likely that, in our model, the main source of IL-17 comes mostly from activated iNKT cells [30], although the contribution of other T cell subsets (i.e. Th17 cells) was not addressed.

A critical role of NK cells in the antitumor immune response was observed since treated mice depleted for NK cells did not survive after 4TOO tumor injection. Interestingly, 4TOO cells express very high levels of the NKG2D ligand retinoic acid early 1 (Rae-1) (data not shown), a molecule directly involved in NK cell activation, which could contribute to the critical role of NK cells in the observed antitumor effect. This is in line with previous studies describing an involvement of NKG2D ligands in the recognition of tumor cells, as part of the immuno editing process [31].

In contrast to some studies [13], CD4^+^ T cells seem to be dispensable for the antitumor effect, likely reflecting the fact that 4TOO cells do not express MHC II molecules [23]. Surprisingly, treated mice depleted of CD8^+^ T cells were still resistant to 4TOO tumor challenge. This suggests that CD8^+^ T cells are not critically involved in the antitumor effect of the Mix + GalCer approach in our model, despite having found a significant amount of tumor-specific IFN-γ secreting CD8^+^ T cells. It is likely that the strong contribution of NK cells to the antitumor effect overcome the potential antitumor effect of those IFN-γ secreting CD8^+^ T cells.

iNKT cells can induce specific B-cell activation [32]. We were able to demonstrate a significant increase of IgG antibodies in Mix + GalCer treated mice that specifically recognize 4TOO tumor cells. Their contribution to the overall antitumor effect has not been addressed; however, since a small percentage of NK-depleted mice were able to resist the tumor challenge, it is tempting to speculate that the tumor specific antibody response may have played a role.

The synthetic glycolipid α-GalCer was tested in several clinical trials with cancer patients demonstrating that it is safe [17]. Despite of this, only transient iNKT cell activation was detected in a minority of patients when α-GalCer was injected alone as a cancer treatment [10, 17]. Other trials were carried out using DC pulsed α-GalCer and showed improved results, suggesting that is important to enhance α-GalCer presentation using DCs [19, 20]. However, in our model, the antitumor effect of Mix + Galcer was consistently better than the approach using α-GalCer-loaded DCs.

## Conclusions

In summary, we show that a combination of DCs and tumor cells pulsed with α-GalCer is a very efficient strategy to induce iNKT activation, with an impressive antitumor therapeutic efficacy as well as a long-lasting and tumor specific immunity, involving T, B and NK cell activation. The use of tumor cells-pulsed DCs combined with iNKT activators represents a highly effective strategy for treating cancer patients.

## Abbreviations

iNKT: invariant natural killer T
DCs: dendritic cells
α-GalCer: alpha-galactosylceramide
IFN-γ: interferon gamma
NK: natural killer
TCR: T cell receptor
APCs: antigen presenting cells
FCS: fetal calf serum
PBS: phosphate buffer saline
GM-CSF: granulocyte–macrophage colony-stimulating factor
Gy: gray
mAb: monoclonal antibody
MFI: mean fluorescence intensity
iv: intravenous
sc: subcutaneous
ip: intraperitoneal
MHC: major histocompatibility complex
IgG: immunoglobulin G
Tregs: regulatory T cells
IL: interleukin
Rae-1: retinoic acid early 1

## References

[1] Terabe M, Berzofsky JA (2008). The role of NKT cells in tumor immunity. Adv Cancer Res.

[2] Vivier E (2012). Targeting natural killer cells and natural killer T cells in cancer. Nat Rev Immunol.

[3] Smyth MJ (2000). Differential tumor surveillance by natural killer (NK) and NKT cells. J Exp Med.

[4] Van Kaer L (2005). alpha-Galactosylceramide therapy for autoimmune diseases: prospects and obstacles. Nat Rev Immunol.

[5] Brennan PJ, Brigl M, Brenner MB (2013). Invariant natural killer T cells: an innate activation scheme linked to diverse effector functions. Nat Rev Immunol.

[6] Cerundolo V (2009). Harnessing invariant NKT cells in vaccination strategies. Nat Rev Immunol.

[7] Fujii S (2002). Prolonged IFN-gamma-producing NKT response induced with alpha-galactosylceramide-loaded DCs. Nat Immunol.

[8] Spada FM, Koezuka Y, Porcelli SA (1998). CD1d-restricted recognition of synthetic glycolipid antigens by human natural killer T cells. J Exp Med.

[9] Smyth MJ (2005). Sequential activation of NKT cells and NK cells provides effective innate immunotherapy of cancer. J Exp Med.

[10] Nieda M (2004). Therapeutic activation of Valpha24 + Vbeta11 + NKT cells in human subjects results in highly coordinated secondary activation of acquired and innate immunity. Blood.

[11] Kobayashi E (1995). KRN7000, a novel immunomodulator, and its antitumor activities. Oncol Res.

[12] Matsuyoshi H (2005). Therapeutic effect of alpha-galactosylceramide-loaded dendritic cells genetically engineered to express SLC/CCL21 along with tumor antigen against peritoneally disseminated tumor cells. Cancer Sci.

[13] Chung Y (2007). An NKT-mediated autologous vaccine generates CD4 T-cell dependent potent antilymphoma immunity. Blood.

[14] Shimizu K (2007). Cross-presentation of glycolipid from tumor cells loaded with alpha-galactosylceramide leads to potent and long-lived T cell mediated immunity via dendritic cells. J Exp Med.

[15] Parekh VV (2005). Glycolipid antigen induces long-term natural killer T cell anergy in mice. J Clin Invest.

[16] Chang WS (2008). Cutting edge: programmed death-1/programmed death ligand 1 interaction regulates the induction and maintenance of invariant NKT cell anergy. J Immunol.

[17] Giaccone G (2002). A phase I study of the natural killer T-cell ligand alpha-galactosylceramide (KRN7000) in patients with solid tumors. Clin Cancer Res.

[18] Schneiders FL (2011). Clinical experience with alpha-galactosylceramide (KRN7000) in patients with advanced cancer and chronic hepatitis B/C infection. Clin Immunol.

[19] Chang DH (2005). Sustained expansion of NKT cells and antigen-specific T cells after injection of alpha-galactosyl-ceramide loaded mature dendritic cells in cancer patients. J Exp Med.

[20] Uchida T (2008). Phase I study of alpha-galactosylceramide-pulsed antigen presenting cells administration to the nasal submucosa in unresectable or recurrent head and neck cancer. Cancer Immunol Immunother.

[21] Ishikawa A (2005). A phase I study of alpha-galactosylceramide (KRN7000)-pulsed dendritic cells in patients with advanced and recurrent non-small cell lung cancer. Clin Cancer Res.

[22] Richter J (2013). Clinical regressions and broad immune activation following combination therapy targeting human NKT cells in myeloma. Blood.

[23] Alvarez E (2010). Dendritic and tumor cell fusions transduced with adenovirus encoding CD40L eradicate B-cell lymphoma and induce a Th17-type response. Gene Ther.

[24] Petersen TR (2010). Potent anti-tumor responses to immunization with dendritic cells loaded with tumor tissue and an NKT cell ligand. Immunol Cell Biol.

[25] Hong S (2013). Tumor cells loaded with alpha-galactosylceramide promote therapeutic NKT-dependent anti-tumor immunity in multiple myeloma. Immunol Lett.

[26] Tatsumi T (2007). Intrahepatic delivery of alpha-galactosylceramide-pulsed dendritic cells suppresses liver tumor. Hepatology.

[27] Eberl G, Brawand P, MacDonald HR (2000). Selective bystander proliferation of memory CD4+ and CD8+ T cells upon NK T or T cell activation. J Immunol.

[28] Galli G (2007). Invariant NKT cells sustain specific B cell responses and memory. Proc Natl Acad Sci USA.

[29] Mattarollo SR (2012). NKT cell adjuvant-based tumor vaccine for treatment of myc oncogene-driven mouse B-cell lymphoma. Blood.

[30] Monteiro M (2013). Induced IL-17-producing invariant NKT cells require activation in presence of TGF-beta and IL-1beta. J Immunol.

[31] Raju S (2016). NKG2D–NKG2D ligand interaction inhibits the outgrowth of naturally arising low-grade B cell lymphoma in vivo. J Immunol.

[32] Tonti E (2009). NKT-cell help to B lymphocytes can occur independently of cognate interaction. Blood.

